# Higher mobility of freshwater mussels in response to unsuitable habitat conditions

**DOI:** 10.1371/journal.pone.0345804

**Published:** 2026-04-15

**Authors:** Andreas H. Dobler, Astrid N. Schwalb, Juergen Geist

**Affiliations:** 1 Aquatic Systems Biology Unit, Department of Life Science Systems, Technical University of Munich, Freising, Germany; 2 Biology Department, Texas State University, San Marcos, Texas, United States of America; Griffith University, AUSTRALIA

## Abstract

Movement behavior of freshwater invertebrates is largely understudied, although gaining insights into behavioral responses may be crucial to better understand their ecology and to guide their conservation. The objective of this study was to examine whether movement distances and burrowing depths of *Unio nanus*, a species of the *U. crassus* complex, and *Anodonta* spp. would vary between three different habitats (dominated by either *U. nanus* or *Anodonta* spp. or in which both species were rare) in a headwater stream in southern Germany within longer (8 weeks) and shorter (48 hours, controlled for substrate) field experiments. We hypothesized that mussels should be the most mobile (i.e., larger locomotion distances) and burrow less in presumably unsuitable habitats where they are rare. Both taxa were most mobile at a sidearm location where both species were rare, and which had the lowest water depth and flow velocity. While *U. nanus* burrowed significantly deeper at the other faster-flowing sites, burrowing of *Anodonta* spp. was more variable and did not significantly differ between sites. The results suggest that low water levels may trigger increased mobility in both species, potentially as an adaptation to avoid drying. The stronger behavioral response of *U. nanus* may reflect its adaptation to more dynamic, lotic environments.

## Introduction

Studies of animal movement have a long history in ecology, and the number of publications has increased substantially over the last two decades, along with the development and use of new technologies [1]. However, most of these studies focus on mammals, fish, and birds, while the behavior of freshwater invertebrates remains largely understudied [1]. Unionid freshwater mussels are a highly imperiled group of organisms that provide crucial ecosystem services [2–5]. At the same time, freshwater mussels of the Unionidae family have severely declined worldwide over decades [6–8]. Their sensitivity to environmental changes makes freshwater mussels excellent indicators for aquatic habitat quality as well as target species of conservation [3,9–11].

Even though freshwater mussels are known to be sedentary, they use their muscular foot to actively move horizontally (locomotion) and vertically (burrowing) [12–14] which can allow them to escape unvafourable conditions. Some mussel species tend to be more mobile than others, which has been associated with life history strategies and their physiological desiccation tolerances [15,16]. Burrowing of mussels can also be triggered by seasonal changes in water temperature, although mussel size (e.g., smaller mussels burrowing deeper) and substrate can also play a role [13,17–20].

Mussel burrowing and crawling behavior is an integral part of their ecology, with particular relevance for their reproduction and survival. For example, some unionid species seasonally move horizontally (forming aggregations; see [18]) and also vertically (surfacing) for reproduction [13,20,21]. In particular, female thick-shelled river mussels (*Unio crassus* complex) have been observed to move to the shoreline during reproduction season to widely spurt their glochidia in water jets, which is assumed to increase host fish attraction and infestation [14,22,23]. Locomotion may also allow mussels to avoid unfavorable conditions [13,16,24,25], and at least some species may actively leave areas with unsuitable substrate to find locations with more suitable substrate [26,27]. In addition to horizontal locomotion (crawling), mussels can also respond to extreme situations such as drought by moving vertically (burrowing) [28]. This behavior can significantly increase their survival but appears to be highly species-specific [15]. Movements away from unsuitable habitat conditions could be used as indicator for habitat suitability for translocated mussels or when mussels from captive breeding are released [26]. Currently, mussel presence or density is usually used as indicator for habitat suitability for translocations, but mussels may be absent especially in re-introductions efforts, for example after restoration of habitat or after a catastrophic event extripated a local population. In that case other indicators such as viability of caged juvenile mussels or potentially movement of adult mussels could be used, if mobility is indeed higher in unsuitable habitat.

The goal of this study was to compare behavioral responses of a lentic (*Anodonta* spp.) and a lotic (*U. crassus* complex) functional mussel group to different habitat conditions. Most mussels in the lentic *Anodonta* spp. group were morphologically clearly identified as *A. anatina*. Since no genetic identification of individual specimens was carried out and since the most abundant *A. anatina* and the rare *A. cygnea* in the study stream can occasionally be morphologically similar, we chose the conservative approach of referring to the lentic group as *Anodonta* spp*.* Potential misidentification of a small number of specimens would not affect the results of the study since its core focus is on comparing the two functional groups (lentic and lotic mussels) which differ markedly in adult body size and shell characteristics, with *Anodonta* spp. growing much larger and having much thinner shells than *Unio crassus* complex.

Mussels of the *Unio crassus* complex are known to occur in streams with higher flow velocity and coarser substrate, but are often found in sites with low shear stress, as shear stress affects juvenile mussels [14,29], and higher amounts of organic matter and fine sediments [30]. In contrast, *Anodonta* spp. mostly occur in standing waters or slow-flowing streams [7]. For this reason, we chose a stream in southern Bavaria where both taxa (*Anodonta* spp. and *Unio nanus*, a species of the *U. crassus* complex [31]) co-occur in a stable community. The location provides a rare opportunity to compare movement behavior of mussels with contrasting habitat preferences under three different habitat conditions at three sites in rather short Euclidean distance (~ 1 km), while sharing similar large scale environmental conditions, such as stream size, discharge, geology, and land cover.

**Fig 1:**
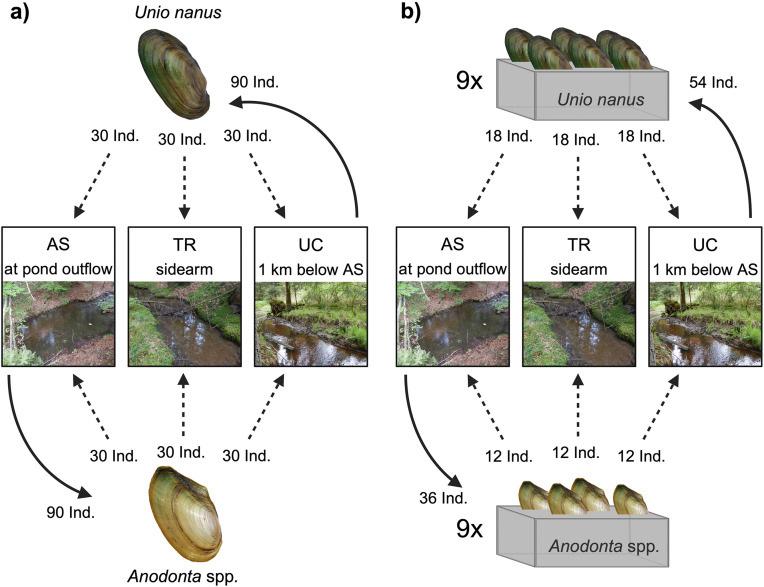
Structure of the two experiments conducted in three habitats: AS (below the pond), UC (1 km downstream of AS), and TR (sidearm). **(A)** Long-term experiment: 90 individuals of *Unio nanus* and *Anodonta* spp. were collected from their dominant habitats (solid arrows), marked with PIT-tags, and divided into three groups. Each group was released into one of the three habitats (dashed arrows). **(B)** Short-term experiment: 54 *U. nanus* and 36 *Anodonta* spp. were collected (solid arrows) and placed in 9 sand-filled boxes (6 *U. nanus* or 4 *Anodonta* spp. per box). Three boxes (containing 18 *U. nanus* or 12 *Anodonta* spp.) were placed in each habitat (dashed arrows).

To address our goal of comparing behavioral responses of lentic and lotic mussels, we examined (1) how locomotion distances (from 24 hours up to 8 weeks) would differ in different parts of a stream with a field experiment translocating mussels into presumably suitable habitat where they were abundant and into less suitable habitat in which they were rare, and (2) burrowing depth of mussels with a shorter experiment (~1day) in which boxes with the same substrate were placed in the same habitats used in the first experiment (Fig 1). We hypothesized that habitat conditions affect the movement behavior of mussels in that mussels (1) move more in unsuitable habitat conditions to avoid unfavorable conditions (Get Out of Town Strategy), and (2) burrow more in suitable habitat conditions (Bunker Down Strategy) to take advantage of the more stable conditions in the sediment (see specific predictions in Table 1). We assumed that higher mussel abundances would indicate suitable habitat and mussels being rare would indicate unsuitable habitat conditions.

**Table 1:** Predicted differences based of hypotheses 1 and 2 (see details in text).

Functional group	Long-term experiment: Locomotion distances		Short-term experiment: Burrowing depth	
	Predicted differences between locations	Results	Predicted differences between locations	Results
*Lotic (U. nanus)*	TR> AS ≥ UC	TR> AS ~ UC	UC ≥ AS> TR	UC ~ AS> TR
*Lentic (Anodonta* spp*.)*	TR ≥ UC> AS	TR > UC ~ AS	AS> UC ≥ TR	AS ~ UC ~ TR

Previous surveys showed that *U. nanus* had the highest density at location UC, lower density at AS, and only a few individuals were found at TR, whereas *Anodonta* spp. had the highest density at location AS, with only a few individuals at UC and TR. See study location in material and methods for more details. All mussels were placed in boxes with sand during the short-term experiment.

## Materials and methods

### Study location and design

The experiments were conducted between 25^th^ April and 21^st^ June 2022 in a headwater stream, the Mooshamer Weiherbach (47°53′25″N, 11°31′0″E), Bavaria, Germany, which supports large populations of both *U. nanus* Lamarck, a species of the *U. crassus* complex [31], and *Anodonta* spp. Almost all individuals of *Anodonta* were clearly identified as *A. anatina*, but conservatively treated at the genus level as *Anodonta* spp. since no genetic identification of individual specimens was carried out and since the most abundant *A. anatina* and the rare *A. cygnea* in the study stream can occasionally be morphologically similar*.* Permission for the field work, including the capture and temporary handling of protected species, was obtained from the District Government of Upper Bavaria (reference no. ROB-55.1–8646.NAT_03-9-10-3).

The study was designed to evaluate the locomotion patterns and burrowing behavior of lotic (*U. nanus*) and lentic mussels (*Anodonta* spp.)*.* under varying environmental conditions. Two complementary experiments were conducted (Fig 1): (1) a long-term (56−57 days) translocation experiment to assess mussel relocation across three distinct habitats and (2) a short-term (17−19 hours) experiment in controlled setups to evaluate burrowing depth. For both experiments, three sites were chosen that differed in their habitat conditions and mussel assemblage composition. The first location (AS), dominated by lentic mussels (*Anodonta* spp.)*,* was located at the outflow of a pond. A mussel survey in the year 2021 (unpublished data) determined the assemblage at this location to consist of 13% (1.4 Ind. m^-2^) *U. nanus* and 87% (9.3 Ind. m^-2^) *Anodonta* spp. of all mussel individuals. The second location (UC, ca. 1,000 m downstream of the pond) was dominated by lotic mussels (*U. nanus*; 99.5%, 102.2 Ind. m^-2^), with only a few individuals of lentic mussels (*Anodonta* spp.). found (0.5%; 0.5 Ind. m^-2^). The third location was a sidearm of the stream (TR, its confluence with the mainstem was 100 m downstream of UC) with lower flow velocity and water depth, where only a few individuals of *U. nanus* and *Anodonta* spp. were present.

In the long-term translocation experiment, mussels were collected from locations where they were dominant (*U. nanus* from the lower stream section and *Anodonta* spp*.* from the upper stream section) and were then relocated to two different habitats (Fig 1A). As a control, a group of mussels from each species was returned to their original habitat, assuming highest suitability of these habitats. In the short-term burrowing depth experiment, mussels were placed in controlled setups consisting of plastic boxes filled with coarse sand (to standardize substrate conditions) that were positioned onto the stream bed at the three locations (Fig 1B).

To characterize the different habitats, we measured stream width and water depths using a measuring tape, turbidity using a handheld turbidity meter Turb 430 IR (WTW, Weilheim, Germany), and flow velocity (m s^−1^) 5 cm below the water surface and 5 cm above ground using a handheld flow meter (Flowtherm NT; Höntzsch, Waiblingen, Germany) at three positions across the stream (left, middle, right) at each site.

In addition, we measured water temperature (°C), dissolved oxygen (mg L^-1^), electrical conductivity (μS cm^-1^, relative to 25 °C), pH value using a handheld Multi 3630 IDS (WTW GmbH, Weilheim, Germany), and redox potential using a handheld pH-meter pH 3110 (WTW, Weilheim, Germany) with a platinum electrode and an Ag/AgCl_2_ reference electrode following Geist & Auerswald [32]. These parameters were measured in the free-flowing water and at 10 cm depth in the substratum.

To characterize the substratum of each habitat, penetration resistance (PR, in kg cm^−2^) was measured using a handheld penetrometer (Eijkelkamp Agrisearch Equipment, Giesbeek, The Netherlands) and sediment samples were taken, fractionated using a wet sieving tower (Fritsch, Idar-Oberstein, Germany) with different mesh sizes (63.0, 20.0, 6.3, 2.0 and 0.85 mm), subsequently dried for 24h at 100 °C and weighed to the nearest g.

During the long-term translocation experiment, average water temperature was about 4.5 °C higher at the location AS (22.1 °C ± 3.9 SD) compared to both UC and TR (Table 2), which is likely due to AS being located directly downstream of the outflow of a large pond, where water warms more strongly and retains heat compared to flowing stream sections. The substrate did not differ drastically between sites, consisting of largely finer sediment (mud and sand) intermixed with some gravel and woody debris from the forest canopy. The highest proportion of fine sediment (53.1%) occurred at AS, while the location UC had the lowest proportion of fine sediment (33.6%, Table 2). Both depth and velocity were lower and conductivity higher at site TR compared to the other sites (Table 2).

**Table 2:** Habitat characterization of the three locations “UC”, “AS” and “TR” with water and sediment parameters.

Parameter	UC	AS	TR
**Water column**			
Temperature (°C)	17.7 ± 3.1	22.1 ± 3.9	17.6 ± 2.7
Conductivity (µS cm^-1^, related to 25 °C)	470.7 ± 33.1	472.7 ± 45.7	570.3 ± 12.3
pH	8.2 ± 0.1	8.2 ± 0.3	8.1 ± 0
Dissolved oxygen (mg L^-1^)	6.7 ± 1.6	6.6 ± 2.4	5.8 ± 1.5
Stream width	1.9	4.3	1.1
Flow velocity (m s^-1^)	0.14 ± 0.14	0.19 ± 0.18	0.09 ± 0.06
Water depth (cm)	10.3 ± 5.2	13.1 ± 5.8	2.2 ± 0.6
Turbidity	2.6 ± 0.0	8.8 ± 0.0	9.6 ± 0.4
Redox-potential (Eh in mV)	228	225	227
**Substrate**			
Amount of grain size <0.85 mm (%)	33.6	53.1	42.0
Amount of grain size 0.85–2.0 mm (%)	5.4	1.6	10.9
Amount of grain size 2.0–6.3 mm (%)	18.2	9.4	21.0
Amount of grain size 6.3–20 mm (%)	42.8	36.0	26.1
Temperature (°C)	11.8 ± 0.2	13.6 ± 0.4	11.4 ± 0.2
Conductivity (µS cm^-1^, related to 25 °C)	573.5 ± 7.4	547.0 ± 8.5	676.0 ± 105.7
pH	7.3 ± 0.1	8.0 ± 0.2	7.7 ± 0.2
Dissolved oxygen (mg L^-1^)	1.8 ± 0.6	1.4 ± 0.4	1.4 ± 0.8
Redox-potential (Eh in mV)	−108.0 ± 43.9	−112.0 ± 14.9	−102.7 ± 17.9
Penetration resistance (kg cm^-2^)	0.6 ± 0.3	1.3 ± 0.7	1.3 ± 0.9

During the short-term burrowing depth experiment, temperature (15.8 to 16.4 °C) and dissolved oxygen concentration (9.7 mg L^-1^) were slightly higher at AS compared to the other two locations (temperature: 13.1 to 13.2 °C at UC; 13.4 to 13.6 °C at TR; and DO: 7.0 mg L^-1^ at TR; 8.5 mg L^-1^ at UC). Flow velocity was highest where the experimental boxes were placed at UC (0.07 to 0.16 m s^-1^), lowest at TR (0.01 to 0.03 m s^-1^), and intermediate at AS (0.01 to 0.07 m s^-1^). The distance to the water surface from the substrate in the experimental boxes was 4–11 cm at UC, 3–8 cm at TR, and 9–18 cm at AS; these depth ranges were within the observed range of habitat conditions or our small study creek.

### Long-term translocation experiment

For the translocation experiment, 90 individuals each of *U. nanus* and *Anodonta* spp. were collected from sections at which the respective species is dominant. All mussels were marked with glue-on shell tags (80 x 40 mm; Hindmarsh Valley, Australia) on the left valve following Hartmann et al. [33] and additionally with 12 mm FDX Passive Integrated Transponder (PIT) tags (APT12 PIT Tag, Biomark, Boise, Idaho, USA) on the right valve. *Unio nanus* were taken from the lower section, while *Anodonta* spp. were taken from the upper part of the stream AS downstream of the confluence with the outflow of the pond.

We randomly distributed all 90 marked individuals of each species into three groups. One group (30 individuals per species) of each species was translocated to the respectively different habitat, one to the nearby sidearm, and one to the original habitat as a control. At each location, three fixed reference points were set around the stream, and the distance between all reference points was measured using a measuring tape (± 0.5 cm). Mussels were placed in small groups of 5–6 individuals, and the initial group location and later the individual position of each detected specimen was determined by triangulation, measuring the distance to each reference point at each time point using a laser measuring device (LaserRange-Master T4 Pro, Laserliner, Arnsberg, Germany). To detect the mussels, a handheld PIT-tag reader (HPR Plus, Biomark, Boise, Idaho, USA) with a portable antenna (BP Plus, Biomark, Boise, Idaho, USA) was used with the power setting adjusted to 60% to pre-screen the stream bed. When a tag was detected, the power was decreased to 20% to narrow down the exact position. Mussel locations were recorded at five different timepoints: 1–2, 6–8, 16–17, 27–28, and 56–57 days after translocation to capture more immediate and potential long-term responses to translocation.

### Short-term burrowing depth experiment

Three plastic boxes (36.5 cm length x 31.0 cm width x 17.5 cm depth) completely filled with coarse aquarium sand (D_50_ = 1.31 mm) were placed on the surface at each of the three locations. The sediment depth allowed all mussels to burrow completely. All boxes were placed on the streambed surface. In total, 54 individuals of *U. nanus* at location UC were tagged with glue-on shell tags on 2^nd^ May 2022, and six individuals were placed in the middle of each box. After 19–23 hours, the location of each mussel was recorded, as well as their burrowing depth as percentage of the shell burrowed (completely at the surface, i.e., 0%, 1–25, 25–50, 50–75–90, and 90–99% of the shell burrowed, and completely burrowed (100%). Similarly, a total of 36 individuals of *Anodonta* spp. were tagged on 3^rd^ May 2022 at location AS, and due to their greater size four individuals were placed in each box. The boxes restricted horizontal movement, and the focus was therefore on burrowing depth. Burrowing depths were recorded 17–19 hours later (on average the position of *Anodonta* spp. was recorded 2.5 hours earlier than those of *U.* *nanus*, but both species had sufficient time to adjust their position, which usually occurs within the first 12 hours after translocation). In addition, their position in the box (or in some cases outside the box) was noted at the end of the short-term burrowing depth experiment.

### Data analysis

Statistical analyses were performed using R (version 4.3.2, Boston, MA). Significance was accepted at p < 0.05 for all statistical analyses. Discovery rate (in %) was calculated for each species, location, and timepoint. The distance mussels moved between the time of recordings was calculated from the cosine theorem using the triangle of the measured length to each reference point and the distance between the reference points. The distance per day was calculated from the measured distance that the mussels moved and the number of days between two sightings. Total locomotion distance was calculated as the sum of the stepwise distances between consecutive observation points for each specimen. To compare the detection rates of the long-term translocation experiment over timepoints, species and their interaction, a repeated measures analysis of variance (ANOVA) was conducted. The analysis was implemented using the ezANOVA function of the R package ez (v. 4.4−0), with timepoint as the within-subject factor and species as the between-subject factor. Time was treated as a categorical factor because measurements were taken at discrete sampling intervals, and the primary interest was in differences between specific timepoints rather than continuous temporal trends. The hierarchical structure of the data, where repeated measurements were nested within individual subjects, was accounted for by defining subjects as unique combinations of species and location. The model included Type III sums of squares to handle unbalanced designs and interactions between factors. Post-hoc comparisons between timepoints were conducted using pairwise t-tests with Bonferroni adjustment for multiple comparisons. To assess the effects of treatment and species on the total locomotion distance of the long-term translocation experiment, a two-way ANOVA with a TukeyHSD posthoc test was performed. The model included square root transformed total locomotion distance as response variable and treatment and species and their interaction as fixed factors. An ANOVA and post-hoc Tukey’s test were used to detect significant differences in burrowing depth for the short-term burrowing depth experiment. Prior to analysis, assumptions of normality and homogeneity of variance were tested to ensure the validity of the ANOVAs. To test the effects of treatment and timepoint of observations on the locomotion distance of the long-term translocation experiment, a zero-inflated generalized linear model with a zero-inflated gamma distribution with log-link function was computed using the glmmTMB function of the R package glmmTMB (v. 1.1.9; [34]). Distance per day was used as response variable and timepoint, species, and location as fixed factors. Interactions between all factors were included.

Because interaction terms involving species were included in the full model, additional models were fitted for each functional group to facilitate interpretation of treatment and time effects within each species. These models included timepoint, location, and their interaction as fixed effects.

Significance levels were assessed with Wald χ^2^ tests using the function ANOVA of the car package (v. 3.1−2; [35]). Pairwise comparisons with adjusted p-values using Bonferroni method were calculated using the emmeans package (v. 1.10.0; [36]). The model was visually evaluated for systematic deviations in the residuals using diagnostic plots provided by the DHARMa package (v. 0.4.6; [37]). This included checks for patterns indicating misspecification, heterogeneity of variances, and potential overdispersion. Unless stated otherwise, results are given as mean ± standard deviation (SD).

## Results

### Mussel morphometry

*U. nanus* specimens, which reach smaller maximum sizes than pond mussels, had a mean shell length of 6.5 cm ± 0.6 SD (n = 54) in the short-term burrowing depth experiment and 5.3 cm ± 0.5 SD (n = 90) in the long-term translocation experiment. *Anodonta* spp. individuals were about twice as large, with individuals in the short-term burrowing depth experiment being, on average, slightly smaller with 10.3 cm ± 2.0 SD (n = 36) than in the long-term translocation experiment (12.0 cm ± 2.0 SD, n = 90). The shell lengths did not differ significantly between the mussel groups randomly placed at the different locations in both experiments (S1 Table; S2 Fig).

### Long-term translocation experiment

**Fig 2:**
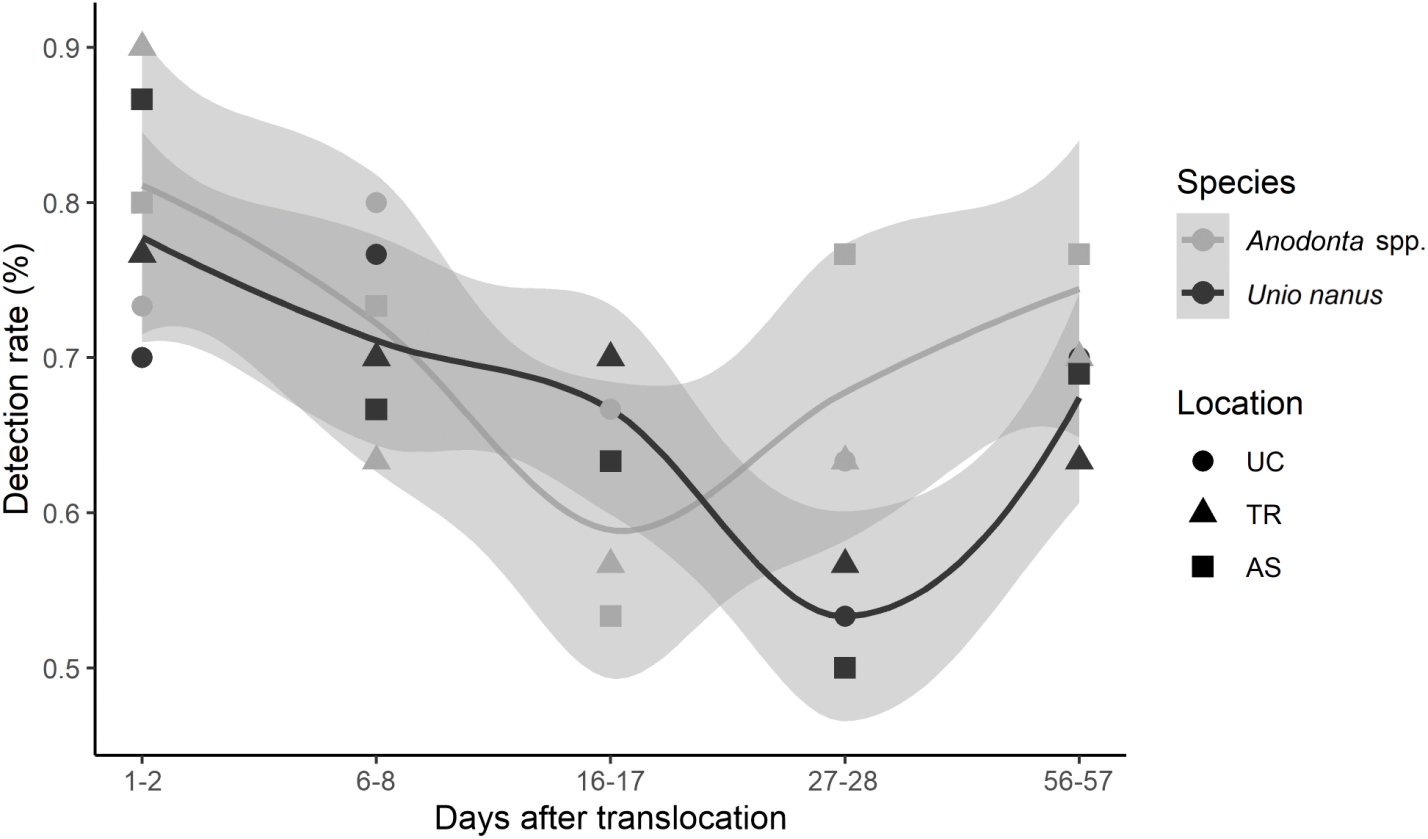
Detection rate (in %) at the different time points of *Unio nanus* and *Anodonta* spp. at each location. Lines indicate the mean detection rate, shaded grey areas the 95% confidence limits.

Throughout the entire experiment, an average of 69.1% of the marked mussels were detected. However, detection rate significantly varied between *U. nanus* (67.3% ± 9.3 SD) and *Anodonta* spp. (70.9% ± 9.8 SD; ANOVA: F_1, 4_= 10.60; *p* < 0.05) as well as between the time points (ANOVA: F_4, 16_ = 7.34; *p* = 0.001). Recapture rates after 1–2 days were significantly higher than at observations 3 (after 16–17 days) and 4 (after 27–28 days; TukeyHSD: *p* < 0.01; see Fig 2). No significant differences in the detection rate were found between the three locations (ANOVA: F_2, 3_ = 0.22; *p* = 0.8).

**Fig 3:**
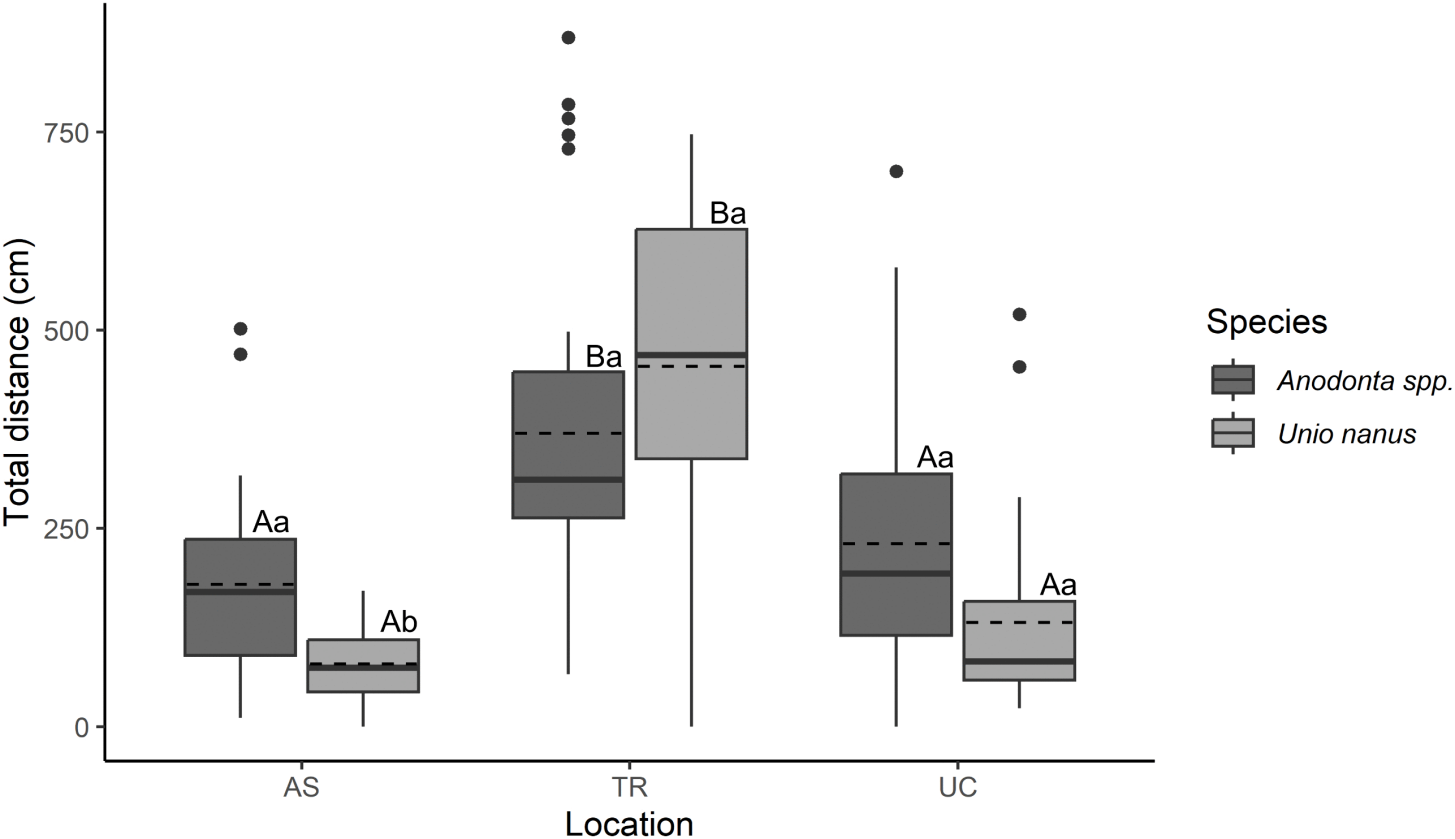
Total distance all *Unio nanus* and *Anodonta* spp. specimens moved at each of the three locations: AS (outflow of the pond, dominated by *Anodonta* spp. ), UC (dominated by *U. nanus*), TR (sidearm, few mussels) during the experiment. Boxplots represent the 25 and 75 percentile with the median marked by a solid horizontal line and the mean indicated by a dashed horizontal line. The dots represent outliers. Different letters above the boxes represent significant differences between the locations (upper cases) and between the species within the same location (lower cases).

Total locomotion distance over the two-month experimental period differed significantly between species and locations (2-way ANOVA: F_1, 174_ = 7.24; *p* < 0.01). Across all sites, mussels moved a mean total locomotion distance of 241 ± 205 cm. The larger *Anodonta* spp. exhibited greater total locomotion distances (260 ± 190 cm) than *U. nanus* (222 ± 218 cm)*.* At site TR, the mean total distance of *U. nanus* (454 ± 209 cm) exceeded that of *Anodonta* spp. (370 ± 218 cm, Fig 3), although this difference was not statistically significant (TukeyHSD; *p* = 0.63).

For *U. nanus*, total locomotion distances were highest at TR, whereas distances at UC and AS did not differ significantly. *Anodonta* spp. showed a similar pattern, with highest total distances at TR, but, contrary to our prediction, distances at AS (where the species was most abundant) were comparable to those at UC, where it was rare (Table 1, Fig 3).

**Fig 4:**
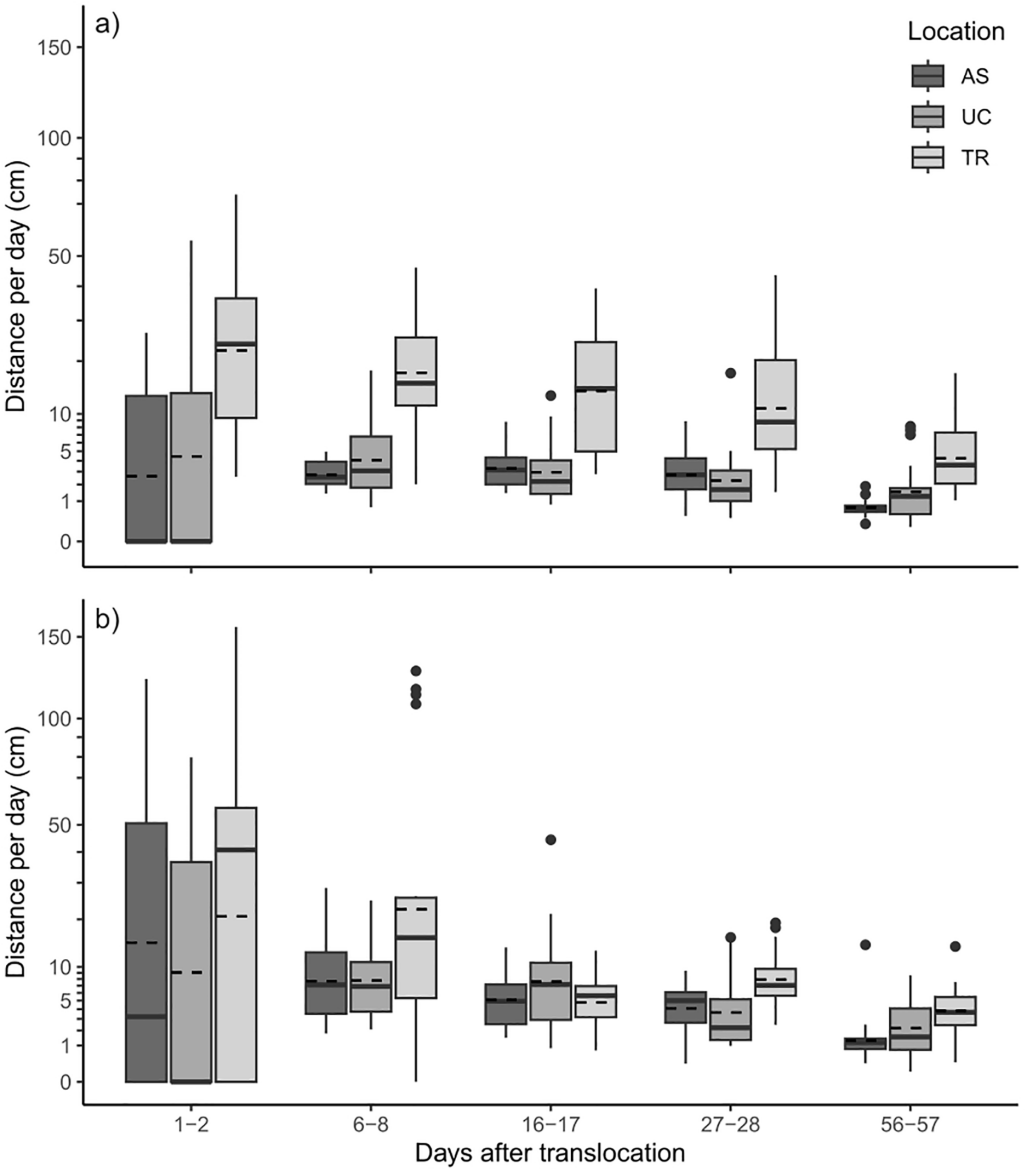
Movement of (A) *Unio nanus* and (B) *Anodonta* spp. individuals at each location between observations. Boxplots represent the 25 and 75 percentile with the median marked by a solid horizontal line and the mean indicated by a dashed horizontal line. The dots represent outliers. Note that the y-axis is square-root scaled.

Similarly, distance per day was significantly higher in *Anodonta* spp. (13 ± 24 cm d^-1^) than in *U. nanus* (8 ± 12 cm d^-1^, model; χ^2^_(1)_ = 50.9; *p* < 0.001; Fig 4), except for location TR*,* at which *U. nanus* consistently moved larger distances than *Anodonta* spp.

Because the three-way interaction between species, location, and timepoint was significant (χ²_(8)_ = 27.5, *p* < 0.001), functional group-specific models were evaluated to clarify patterns within each species: For *U. nanus*, daily locomotion distance varied significantly with timepoint (χ²_(4)_ = 230.4, *p* < 0.001), location (χ²_(2)_ = 216.3, *p* < 0.001), and their interaction (χ²_(8)_ = 37.5, *p* < 0.001; Table 3). The largest daily movement distances were observed at TR, while UC and AS did not differ substantially (Fig 4A). For *Anodonta* spp., daily locomotion distance was significantly affected by timepoint (χ²_(4)_ = 431.6, *p* < 0.001), location (χ²_(2)_ = 34.7, *p* < 0.001), and their interaction (χ²_(8)_ = 43.4, *p* < 0.001). Highest daily movement occurred at TR, whereas daily distances at AS remained similar to those at UC despite higher local abundance (Fig 4B).

**Table 3:** Results of species-specific zero-inflated generalized linear models examining differences in distance moved per day between three locations, five different timepoints, and the interactions between location with timepoint.

Response variable	Factors	χ^2^	*df*	*p*	χ^2^	*df*	*p*
		*Unio nanus*			*Anodonta* spp*.*		
Distance per day	Location	216.3	2	<0.001	34.7	2	<0.001
	Timepoint	230.4	4	<0.001	431.6	4	<0.001
	Location*timepoint	37.5	8	<0.001	43.4	8	<0.001
*R* ^2^		0.38			0.29		

Daily locomotion decreased over the course of the experiment for both species. In *Anodonta* spp., mean movement declined from 30 ± 37 cm d ⁻ ¹ at the first observation to 13 ± 24 cm d ⁻ ¹ at the last two timepoints, while in *U. nanus* it decreased from 14 ± 17 cm d ⁻ ¹ to 8 ± 12 cm d ⁻ ¹. Species-specific models confirmed that these declines were statistically significant across most timepoints (*U. nanus*: χ²_(4)_ = 230.4, *p* < 0.001; *Anodonta* spp.: χ²_(4)_ = 431.6, *p* < 0.001; Table 3), with Bonferroni-adjusted pairwise comparisons showing no significant differences only between intermediate timepoints (6–8 days vs. 16–17 days and 16–17 days vs. 27–28 days for *U. nanus*; 16–17 days vs. 27–28 days for *Anodonta* spp.; *p* = 1.00).

Variation in locomotion was highest during the first two days, particularly for *Anodonta* spp. (range: 0–157 cm; coefficient of variation: 122%). In contrast, daily differences between locations for *Anodonta* spp. became negligible after approximately two weeks (range: 0–44 cm; coefficient of variation: 101%; Fig 4).

### Short-term burrowing depth experiment

**Fig 5:**
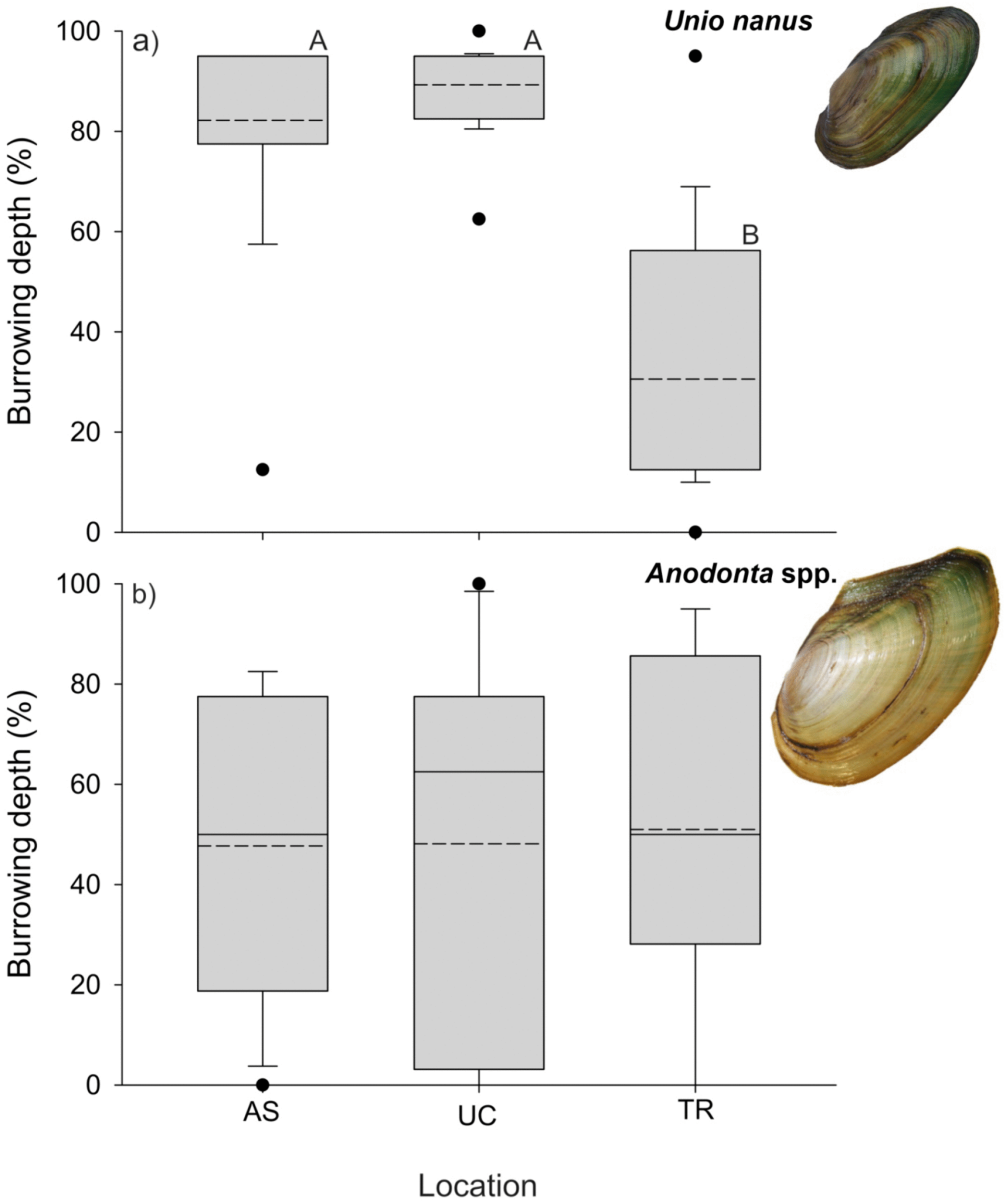
Burrowing depth, measured as percentage of the shell burrowed (completely at the surface, i.e., 0%, 1-25, 25-50, 50 to 75-90, and 90-99% of the shell burrowed, and completely burrowed (100%)) of (A) *Unio nanus* and (B) *Anodonta* spp. in the short-term experiment at three different locations. Boxplots represent the 25 and 75 percentile with the median marked by a line and the mean indicated by a dashed line. The dots represent outliers. Different letters above boxes represent significant differences between locations (Tukey test *p* < 0.05).

For *U. nanus*, at the location near the pond (AS) and the habitat of *U. nanus* (UC), burrowing depth in all experimental boxes was on average ≥ 80%, and the majority of the mussels (usually 4–5 of the 6 mussels) stayed in the middle of the box. Those that did move tended to be found at the upstream edge of the box. In contrast, at the site with the lower flow velocity and lower water depth (TR), mussels were only burrowed on average 25–35% in all 3 experimental boxes, which was significantly lower compared to the other sites (F_2, 50_ = 43.1, *p* < 0.001, Fig 5). There were no significant differences between boxes (F_2, 48_ = 0.1, *p* = 0.9). TR was the only site at which all mussels had either moved to the edge of the box or had even “escaped” and moved mostly upstream of the box (4 individuals, 1 of which could not be recovered).

For *Anodonta* spp., burrowing depth varied more widely between individuals, and there were no significant differences between locations (F_2, 31_ = 0.03, *p* = 0.97, Fig 5). However, two individuals were found downstream and outside of the box at the location with the highest flow velocity (UC), and another 3 individuals moved outside the box at location TR (two of them could not be recovered).

## Discussion

The results of our experiment confirm our hypothesis (1) that both mussel species move more in unsuitable habitat conditions to avoid unfavorable conditions, i.e., the *Get Out of Town Strategy.* Hypothesis (2) that mussels would burrow more in suitable habitat conditions, i.e., the *Bunker Down Strategy* (presumably to take advantage of the more stable conditions in the sediment) could only be confirmed for the lotic mussels (*U. nanus*), but not for the lentic mussels *(Anodonta* spp.). However, no significant differences were detected between the other two suitable habitats in which one species dominated or co-occurred with the other species. Differences in relative mussel abundances in these habitats were most likely driven by conditions that did not occur during our study or that were rather driven by mass effects (dispersal from a nearby source population), which is in contrast to our assumption we used for our predictions that mussel abundances would indicate habitat suitability. For instance, *Anodonta* may be more likely dislodged during flooding (see discussion below). In addition, the pond does not provide habitat for *U. nanus*, but an ideal source population for *Anodonta,* that could explain their dominance immediately downstream of the pond.

In addition, there were differences in the responses to unsuitable habitat between species for both locomotion and burrowing behavior. For *Anodonta*, differences in movement distances between sites diminished after the first week and no significant differences in burrowing depths were found between sites. In contrast, *U. nanus* showed consistently higher movement distances at the unsuitable habitat site (TR) throughout the entire 8-week study period, as well as pronounced differences in burrowing depths between sites. In general, *U. nanus* appeared to be more mobile, not just in terms of burrowing, but also for horizontal movements, as locomotion per cm shell length was almost twice as high for *U. nanus* (4.2 cm ± 4.2 SD) than for *Anodonta* spp. (2.2 cm ± 1.6 SD).

Our findings of higher mobility being triggered by unfavorable habitat conditions build on previous studies that examined movement of freshwater mussels in response to different (adverse) habitat conditions, such as dewatering [15,16], rising water levels [13], substrate composition [26,27], and water depth [38]. Water depth and velocity have been previously suggested as key habitat parameters indicating habitat suitability for *U. nanus* [14,38,39]. This is also in line with the observed strong microhabitat preference of radio-tacked individuals dispersing over large area and stabilizing their position at ~20 cm water depth [38]. Thus, a higher mobility of *U. nanus* at the presumed unsuitable habitat (TR), characterized by the lowest velocity and shallowest water depth, was expected based on our hypothesis and species-specific habitat preferences.

Similar to *U. nanus,* larger locomotion distances of *Anodonta* spp*.* (at least within the first week), and escape movements of a few individuals outside the experimental boxes were observed at TR*.* In contrast to *U. nanus, Anodonta* spp. is considered a more lentic species, and high abundances can be found in habitats with low or no flow [40]. Therefore, it is unlikely that low flow velocity, but rather the low water depth, may have triggered higher mobility at TR. A response to low water depth could be an adaptation of both species to escape and cope with drying of parts of the water body with higher mobility, allowing the mussels to find deeper water even if by random chance and not by directed movement [38].

The higher locomotion of *U. nanus* at location TR compared to *Anodonta* spp. in the long-term translocation experiment, despite *Anodonta* showing higher locomotion at the other sites, suggests a stronger response of *U. nanus* to unsuitable habitat conditions, which may be typical for lotic species in disturbance-prone systems. *U. nanus* often occur in smaller streams and channels, sometimes less than 1 m wide, which can be more prone to larger and faster relative water level fluctuations than lakes, in which *Anodonta* spp. tends to be most abundant. In addition, we found that locomotion of *U. nanus* continued to be significantly higher throughout the study period, which is in contrast to other movement studies in which locomotion of mussels decreased over time when they were placed in suitable habitat [38]. Consequently, such information is crucial for reintroductions along river longitudinal profiles [29].

In contrast to our prediction, *Anodonta* spp. was not more mobile at the location dominated by *U. nanus* (no difference between UC and AS)*.* This could be due to higher flow velocity making movement more difficult or mussels may avoid to move to areas where they could be dislodged [38]. Alternatively, higher flow velocity may not necessarily be detrimental for *Anodonta* during normal flow conditions, although their larger size and their narrow, wedge-shaped, shells may make them more prone to dislodgement during flooding than smaller thick- and round-shelled *U. nanus*. A few individuals were found downstream of the experimental box together with some sand at UC where higher local velocities occurred, which is unlikely indicating active escape behavior, but passive dislodgement. Differences in locomotion distances and burrowing depth between locations could also be explained by differences in substrate, as this was shown for another lotic species, *Margaritifera margaritifera* [26,41], however, the results of our short-term burrowing depth experiment, in which substrate was the same at all three locations, suggest that this does not apply to the species tested here.

Burrowing depth of *Anodonta* spp. showed larger variation, and no significant differences between locations were found. Shell size may play a key role for burrowing as smaller individuals are known to burrow deeper than larger ones [13,17]. *Anodonta* spp. used in our experiments had larger shell sizes and also a larger variation in shell size compared to *U. nanus*, which may have resulted in larger variation in burrowing depth and less burrowing of *Anodonta* compared to *U. nanus,* being in line with previous observations [25]. Morphologically most individuals (in both experiments) were identified as *A. anatina*, but since the morphological differentiation between *A. anatina* and *A. cygnea* can be difficult in some habitats [42], a contribution of *A. cygnea* cannot be fully excluded. Consequently, behavioral patterns observed for *Anodonta* spp. should be interpreted at the genus (functional group) level rather than as species-specific responses, and part of the observed variation may reflect interspecific differences within the genus [27]. Translocation can affect mussel behavior [43] and likely caused the high mobility detected in the first 1–2 days of the long-term experiment. However, all tagged mussels were handled and two thirds were translocated to two different sites. Thus, if translocation effect caused higher mobility at TR, this should have also occurred at the other site to which they were also translocated, but this was not observed. A limitation in our study is that each habitat type was represented by a single site and future studies should test the generality of these patterns.

Conservation measures such as translocation of adult mussels or re-stocking of juveniles are increasingly used to support mussel populations [44–46], but they require careful habitat selection [47]. Relying solely on mussel presence or abundance can be misleading, as individuals may occur in unsuitable sink habitats due to source-sink dynamics. For instance, in this study, individuals of *Anodonta* spp. found at TR in our study were likely flushed out via infested host fish from the upstream pond with a large population of *Anodonta*, suggesting the pond functions as a source habitat while TR may represent a sink habitat*.*

Using mussel mobility as an indicator of habitat suitability could complement multi-endpoint frameworks that combine physiological, ecological, and environmental indicators to assess freshwater mussel translocation success, which have been shown to provide a more robust assessment than single metrics alone [43,48]. However, mobility as an indicator has several limitations, as movement patterns only reflect conditions during time of observation, movement can be restricted by substrate, and individual variation may obscure general patterns [49,50]. Therefore, if mobility is used as an indicator for habitat suitability, it should always be applied in combination with other considerations (e.g., viability of cages juvenile mussels, or if mussels are present, identification of healthy mussel beds comprising individuals of various sizes and ages can thus also be a valuable indicator for high habitat quality and stability).

## Supporting information

S1 Table Shell dimensions of the specimens of *Unio nanus* and *Anodonta* spp. used in the long-term experiment. (DOCX)

S2 Fig Shell length of used *Unio nanus* and *Anodonta* spp. individuals in the (I) long-term and (II) short-term experiment at three different locations. Boxplots represent the 25 and 75 percentile with the median marked by a line and the mean indicated by a dashed line. The dots represent outliers as defined by the 1.5 times of the interquartile range (IQR).
(TIF)
