# Supplementary material for: Higher mobility of freshwater mussels in response to unsuitable habitat conditions
Source: PLoS One. 2026 Apr 15;21(4):e0345804. doi: 10.1371/journal.pone.0345804 (PMC13082599; doi:10.1371/journal.pone.0345804)
Supplement: S1 Table — (DOCX) [file pone.0345804.s001.docx]

S1 Table Shell dimensions of the specimens of *Unio nanus* and *Anodonta* spp. used in the long-term experiment.

| Location | Shell lenght (cm) | Shell width (cm) | Shell height (cm) |
| --- | --- | --- | --- |
| *Unio nanus* |  |  |  |
| UC | 5.2±0.3 | 2.0±0.2 | 3.0±0.2 |
| TR | 5.3±0.5 | 2.0±0.2 | 3.0±0.2 |
| AS | 5.3±0.7 | 2.1±0.3 | 3.0±0.3 |
| *Anodonta* spp. |  |  |  |
| UC | 11.8±1.7 | 4.0±0.9 | 6.3±0.8 |
| TR | 11.9±2.3 | 3.9±1.0 | 6.3±1.1 |
| AS | 12.2±2.1 | 4.0±0.7 | 6.5±1.1 |
